# Association Between Medication Literacy and Medication Adherence Among Patients With Hypertension

**DOI:** 10.3389/fphar.2019.00822

**Published:** 2019-07-19

**Authors:** Shuangjiao Shi, Zhiying Shen, Yinglong Duan, Siqing Ding, Zhuqing Zhong

**Affiliations:** ^1^Nursing Department, Third Xiangya Hospital, Central South University, Changsha, China; ^2^Xiangya School of Nursing, Central South University, Changsha, China

**Keywords:** medication literacy, medication adherence (MeSH), hypertensive patients, association, blood pressure control

## Abstract

**Background:** Few studies have investigated the association between medication literacy and medication adherence as well as the influence of medication literacy on medication adherence in hypertensive patients. Thus, the goal of the present study was to determine the association between medication literacy and medication adherence in hypertensive patients.

**Methods:** A cross-sectional survey was conducted between August 2016 and December 2016. Self-administered questionnaires were completed, including a self-developed and structured socio-demographic questionnaire; a self-developed, validated, and self-reported Medication Literacy Scale for Hypertensive Patients (C-MLSHP) used for medication literacy measurement; and the Chinese Version of the Morisky Medication Adherence Scale-8 (C-MMAS-8), an eight-item validated, self-report scale for adherence measurement with a total score range of 0–8. A cut-off of 6 was applied to differentiate adherence levels, including patients with an MMAS score <6 (low adherence), MMAS score = 8 (high adherence), and MMAS score ≥6 and <8 (moderate adherence). In this study, hypertensive patients’ medication literacy levels and adherence to antihypertensive agents were identified. Pearson correlation analysis was carried out to identify the correlation between medication literacy and adherence. Binary logistic regression analysis was performed with medication adherence as the outcome variable in order to confirm factors associated with medication adherence.

**Results:** A total of 420 hypertensive patients, including 198 women and 222 men with a mean age of 60.6 years (SD = 12.4), were recruited. The mean score of hypertensive patients on the medication literacy scale was 24.03 (SD = 5.13). The mean scores of the four dimensions of knowledge, attitude, skill, and behavior on the medication literacy scale of this study were 6.22 ± 2.22, 5.04 ± 1.16, 4.50 ± 2.21, and 8.27 ± 1.90, respectively. Regarding medication adherence, the mean score of the C-MMAS-8 in this study was 4.82 (SD = 2.11). A total of 63.6% of patients presented with low adherence, 29.5% presented with moderate adherence, and 7.6% presented with high adherence. The Pearson correlation results showed that medication literacy (r = 0.342, P < 0.01) as a whole variable and the three dimensions of knowledge (r = 0.284, P < 0.01), attitude (r = 0.405, P < 0.01), and behavior (r = 0.237, P < 0.01) were significantly associated with medication adherence. Binary logistic regression analysis indicated that annual income [OR 1.199 (95% CI: 1.011–1.421); P = 0.037] and two dimensions of attitude [OR 2.174 (95% CI: 1.748–2.706); P = 0.000] and behavior [OR 1.139 (95% CI: 1.002–1.294); P = 0.046] in medication literacy were found to be independent predictors of medication adherence. Individuals with better attitudes and behavior literacy in medication literacy were more likely to adhere to the use of antihypertensive agents. Those who had higher annual incomes were more likely to adhere to the use of antihypertensive agents.

**Conclusion:** The levels of medication literacy and medication adherence of hypertensive patients are suboptimal and need to be improved in China. The level of medication literacy in patients with hypertension could affect their adherence to antihypertensive drugs. It was suggested that hypertensive patients’ medication adherence could be improved and driven by increasing the medication literacy level, especially in the attitude and behavior domains. Pertinent strategies that are specific to several dimensions of medication literacy should be developed and implemented in order to promote full medication literacy among hypertensive patients, thus facilitating optimal adherence and blood pressure control.

## Introduction

Hypertension has become one of the leading burdens of disease and a public health problem globally (Irazola et al., 2016). A review and analysis study showed that the proportion of individuals with an elevated systolic blood pressure of 140 mmHg or higher increased from 17,307 to 20,526 per 10,000 over the past 25 years (Forouzanfar et al., 2017) and is still increasing. Furthermore, its prevalence was projected to increase by 30% by the year 2025 (Lim et al., 2012; Abegaz et al., 2017). According to the “Report on Disease of Cardiovascular in China 2017” (Chen et al., 2018), there were 270 million patients in China diagnosed with hypertension, which was at the top of the list of all cardiovascular diseases. Uncontrolled blood pressure and constantly progressive hypertension can eventually evolve to become severe complications and morbidities. Cardiovascular diseases are the leading cause of premature death worldwide (Woolf et al., 2018; Zhao et al., 2019). Direct expenditure on treatment for hypertension has accounted for 6.61% of the total healthcare expenditure in China. Nevertheless, the rates for hypertension awareness, treatment, and control in China are still only 46.5%, 41.1%, and 13.8%, respectively (Chen et al., 2018). The low percentage of patients with controlled blood pressure has been a great concern in many other countries (Ofili et al., 2015), though great efforts and costs have been invested in addressing the problem (Irazola et al., 2016; Forouzanfar et al., 2017; Jafar et al., 2018; Sarfo et al., 2018).

Medication adherence can be defined as the extent to which one’s medication-taking behavior follows that which is mutually agreed upon by the prescribing physician (Sabaté, 2003). For patients with hypertension, lifetime and persistent antihypertensive therapy is one of the most effective ways to achieve ideal blood pressure levels (Sabaté, 2003). In addition, it has been confirmed that suboptimal blood pressure control is closely associated with patients’ poor adherence or nonadherence to antihypertensive drugs (Morisky et al., 2008; Abegaz et al., 2017; Al-Noumani et al., 2018; Beune et al., 2019; Uchmanowicz et al., 2019). Patients with poor adherence were found to be at 1.417 times more likely to have uncontrolled hypertension than those with good adherence (Xue et al., 2019). Therefore, good adherence to antihypertensive agents is considered critical for patients to achieve optimal blood pressure control. However, undesirable levels of medication adherence to therapy for hypertensive patients have been a major public challenge globally (Abegaz et al., 2017; Nielsen et al., 2017; Rampamba et al., 2018). The pooled percentage of non-adherence to hypertensives ranges from 45.2% to 66.7% in low- and middle-income countries (Abegaz et al., 2017; Nielsen et al., 2017; Boima et al., 2015). Furthermore, only 6.2% of hypertensive patients were confirmed to have a high level of adherence in Saudi Arabia (Fatani et al., 2019). In China, only 21.3–35.23% of patients showed good or optimal adherence to their antihypertensive therapy (Wong et al., 2015; Hou et al., 2016; Ma, 2016; Pan et al., 2017). By contrast, patients from Western countries tend to have higher adherence (Uchmanowicz et al., 2019). Several factors associated with poor or non-adherence have also been identified in many recent studies. These include socio-demographic factors (Abegaz et al., 2017; Nurhussein et al., 2018; Fatani et al., 2019; Uchmanowicz et al., 2019), such as gender, age, education level, occupational status, or even race; socio-economic status, such as annual income and medical insurance (Boima et al., 2015; Nielsen et al., 2017); and family disease history, number of prescribed drugs, comorbidity, and duration of hypertension as clinical disease-related factors (Choi et al., 2018; Williams et al., 2018; Uchmanowicz et al., 2019). Furthermore, psychosocial factors also influence medication adherence, such as depressed emotion, perceived severity of disease, self-rated health, perceived symptoms, and self-efficacy (Al-Noumani et al., 2018; Asgari et al., 2019). Knowledge of hypertension and patients’ literacy (Boima et al., 2015; Shirindi et al., 2016; Pan et al., 2017) were also found to be predictors of medication adherence. There have been interventions and measures pertinent to hypertensive patients’ adherence improvement, and certain interventions have been confirmed to be effective. These include implementing fixed-dose combination (FDC) therapy (Du et al., 2018), a pharmacist-led educational program on patients’ knowledge of medications (Gwadry-Sridhar et al., 2013; Amer et al., 2018), health promotion, and appointment reminders as well as medication-taking through smartphone applications as a reminder and disease education resource (Contreras et al., 2019), multicomponent practice-based health coaching (Wu et al., 2018), booklets and blood pressure tracking (Dawes et al., 2010), collaborative prescribing, simplified medication regimens, sufficient communication with patients, medication adherence-improving aids or behavioral support, and appropriate follow-up (Jimmy and Jose, 2011). However, these interventions focus on facilitating medication-taking, offering reminders, or imparting disease and medication knowledge. Some of these approaches do not address the essential underlying factors behind patients’ nonadherence, while others are too monotonous, concentrating only on knowledge enrichment and not being comprehensive. Therefore, shedding light on solutions to address medication adherence issues must be attempted to achieve optimal adherence and blood pressure control.

An international consensus on the definition of medication literacy is the degree to which individuals can obtain, comprehend, communicate, calculate, and process patient-specific information about their medication to make informed medication and health decisions in order to safely and effectively use their medications regardless of the mode by which the content is delivered (e.g., written, oral, and visual) (Pouliot et al., 2018). It is health literacy in the context of medication use. Our research team conducted a preliminary study on the exploration of the connotation and theoretical construction of medication literacy, which showed results consistent with the international definition consensus on medication literacy. There are four dimensions of knowledge, attitude, skill, and behavior covered by medication literacy that were extracted from its definition and connotation through our theoretical research on medication (Zhong et al., 2016; Pouliot et al., 2018); every dimension is key to ensuring patients’ safe use of medication. Correct and adequate knowledge about hypertension and its medication treatment along with positive perceptions and attitudes toward disease therapy for hypertensive patients is fundamental to safe medication use and persistent adherence (Jande et al., 2017; Al-Noumani et al., 2018; Amer et al., 2018). In addition, appropriate skills to calculate and process patient-specific information about their medications are prerequisites for making informed medication and health decisions to achieve safe and effective medication behavior (Foster et al., 2018). Inadequate medication literacy levels have been reported by related studies for other populations (Zhong et al., 2016; Zheng et al., 2017; Cordina et al., 2018). However, to the best of our knowledge, there is a paucity of studies assessing medication literacy for hypertensive patients. In addition, lower medication literacy was found to be associated with inappropriate medication-taking behavior (Lee et al., 2017; Piran et al., 2017). Since health literacy has been identified to be the direct or indirect predictor of medication adherence and blood pressure control (Shibuya et al., 2011; Nafradi et al., 2016; Wannasirikul et al., 2016), we speculate that medication literacy, as in health literacy with regard to the process of medication use, may be associated with medication adherence.

However, there is a lack of scientific evidence regarding the association between the level of medication literacy in hypertensive patients and adherence to their medication as well as the effects of medication literacy on medication adherence.

This study therefore aimed to determine whether and how the level of medication literacy among hypertensive patients affects their medication adherence in order to develop pertinent promotion strategies to improve hypertensive patients’ medication adherence and better control their blood pressure and disease state. In addition, this study aimed to provide insight on how to manage the medication adherence of hypertensive patients.

## Materials and Methods

### Design and Setting

A cross-sectional survey was carried out in Yuelu District of the city of Changsha, which is located in the southern part of China, from August to December 2016. A stratified multi-stage cluster sampling was used to select the targeted investigation settings. In China, all hospitals and healthcare services are classified into three levels in terms of institution scale, academic orientation, specialties, and technical facilities. There are 15 tertiary hospitals, 10 secondary hospitals, and 15 primary healthcare services in Yuelu District of Changsha, China. Stratified cluster sampling was used with a stratified sampling ratio of 20% to randomly select three tertiary hospitals, two secondary hospitals, and three community healthcare services. Hypertensive patients attending the outpatient clinic setting of these selected hospitals or healthcare services were recruited.

### Participants

Patients were eligible to participate in this study if they met the following inclusion criteria: patients were diagnosed as hypertensive according to the 2011 prevention and treatment guidance for hypertension in China (Chen et al., 2018), which is systolic BP≥140 mmHg and/(or) diastolic BP≥90 mmHg; patients were ≥18 years old; patients had been on antihypertensive treatment for at least 2 weeks, including both newly treated hypertensive patients and those who were already on antihypertensive medication for a longer period of time; and patients could understand what others said—namely, they could speak Chinese and communicate well with others.

Subjects were excluded if they had any of the following conditions: severe or acute hypertension or other unstable and uncontrolled cardiovascular and cerebrovascular diseases; psychological and mental illness or pharmacotherapy for mental health conditions; hearing and communication disabilities; dementia or cognitive impairment; cancer; New York Heart Association Class III or IV heart failure, or unstable angina; or severe disease of other organs or systems.

## Methodological Details and Procedures

Hypertensive patients who were eligible were invited to participate in the study and provided with information on the study objectives, study content, and investigation procedures as well as the principle of anonymity of this study. Upon agreement to participate in the study, informed consent forms were signed. Questionnaires were then given and completed by participants (it took approximately 15 min to complete the questionnaires) during their time waiting for clinic consultation or after the clinic visit in the waiting room of the outpatient clinic. A total of 420 hypertensive patients who visited physicians during the investigation period were enrolled and completed these questionnaires. Trained master’s degree students were recruited to distribute and collect questionnaires in the Chinese language. For illiterate participants, the researchers read the question items word by word exactly as they appeared on the questionnaires. Responses were recorded on the questionnaire. The questionnaires were collected immediately after being completed, checked for any missing information, and followed up with the participants.

This study was reviewed and approved by the Ethics Committee of the Third Xiangya Hospital, and permission was granted by the managers of each investigation unit.

## Measurements

### Demographic Questionnaire

This was a self-developed, structured, and self-administered questionnaire used to obtain information on sociodemographic variables, such as gender, age, level of education, annual income, marital status, occupational status, registered family residence, type of medical insurance, family history of hypertension, complications of hypertension (comorbid conditions), number of antihypertensive drugs prescribed, living conditions, and the number of persons in the household.

## Chinese Medication Literacy Scale for Hypertensive Patients (C-MLSHP)

This is a validated and self-administered Medication Literacy Scale for Hypertensive Patients, which was self-developed by our research team in a prior study. There are four dimensions of knowledge, attitude, skill, and behavior included, along with 37 items. The knowledge dimension covered 9 items, the attitude dimension included 8 items, the skill dimension included 7 items, and the behavior dimension comprised 13 items.

Cronbach’s α coefficient was 0.849 for the full scale and ranged from 0.744 to 0.783 for the dimensions. The split-half reliability of the whole scale was 0.893, and for each dimension, it ranged from 0.793 to 0.872. The test–retest reliability of the whole scale was 0.968, and for each dimension, it ranged from 0.880 to 0.959. Therefore, good reliability of this self-developed questionnaire was confirmed in our prior study for scale development. For the Chinese Medication Literacy Scale for Hypertensive Patients (C-MLSHP), the whole scale and all of the items showed a content validity index above 0.8 (the content validity index for the whole scale of 0.968; the content validity index for the items was in the range of 0.833–1.000), indicating that the content validity of the scale and all items were at an acceptable level. The scores for the 37 items were summed to create an overall medication literacy score ranging from 0 to 37, with higher scores indicating a higher medication literacy level. For items with the dimensions of knowledge and skills, a correct answer for one item is scored 1, and a wrong answer is scored 0. A five-point Likert response was used for items in the dimensions of attitude and behavior, in which scores of 1.0, 0.75, 0.5, 0.25, and 0 were applied. In addition, five items in the attitude dimension and one item in the behavior dimension were reverse scored.

As for the studied population of hypertensive patients in this present study, acceptable reliability and validity were also found for the C-MLSHP. Cronbach’s α coefficient was 0.826 for the full scale, 0.802 for the skill dimension, 0.731 for the behavior dimension, 0.793 for the attitude dimension, and 0.734 for the knowledge dimension.

## Chinese Version of the Morisky Medication Adherence Scale-8 (C-MMAS-8)

The original version of MMAS-8 was developed by Morisky and their research team (Morisky et al., 2008), and it was a simple, practical, and cost-effective self-report assessment tool to evaluate patients’ medication adherence. The eight-item medication adherence scale showed good reliability and validity for assessing adherence in patients with hypertension, with a Cronbach’s alpha coefficient of 0.83 (Morisky et al., 2008). In addition, it was significantly associated with blood pressure control (Morisky et al., 2008). In this scale, the response categories are yes/no for seven items with dichotomous response options and a five-point Likert scale response option for the last item. These items provide information about the barriers to medication adherence, such as forgetting to take medication, not taking medication when one feels worse, and having difficulties in complying with a treatment regimen. The scores for the eight items were summed to create an overall adherence score ranging from 0 to 8, with higher scores indicating better adherence. The recommended cutoff point of 6 was used, as suggested by Morisky et al. (2008). An MMAS score <6 indicated low adherence, a score = 8 was considered high adherence, and a score ≥6 and <8 indicated moderate adherence (Morisky et al., 2008).

In a previous study (Morisky et al., 2008), this scale’s sensitivity to identify poor vs. good adherence was estimated to be 93%, and its specificity was 53%, thus indicating acceptable levels of sensitivity and specificity in detecting nonadherence.

A Chinese version of the MMAS-8 (Yan et al., 2014) (C-MMAS-8) was used in the present study. It was validated in a group of myocardial infarction patients, and the results showed good reliability and good validity (Cronbach’s α = 0.77, and pretest and post-test correlation coefficient of 0.88). In the group of the studied Chinese hypertensive patients in the present study, Cronbach’s α coefficient was 0.716, which was acceptable.

### Data Analysis

All of the collected data were entered into SPSS 23.0 and analyzed. All continuous variables with normal distribution were described in means and standard deviation (mean ± SD), and the categorical variables were summarized by numbers or percentages. Pearson correlation analysis was used to determine the correlation between medication literacy and medication adherence. Patients with low and moderate adherence were categorized as poor adherers, and those with high adherence were categorized as good adherers. Therefore, socio-demographic characteristics were calculated for participants with poor and good medication adherence. All variables listed above coupled with the four dimensions of medication literacy—namely, knowledge, attitudes, skills, and behavior—were consecutively tested for a significant association with medication adherence as the dependent variable in the binary logistic regression analysis. All statistical tests used were two-tailed, and P-values < 0.05 were considered statistically significant.

## Results

### Patient Characteristics

In this study, a total of 450 questionnaires were distributed, of which 420 were completed, yielding a response rate of 93.33%. The sociodemographic characteristics of the 420 participants in the study are presented in **Table 1**. The mean age of the respondents was 60.6 years (SD = 12.4 years), with 59.5% over 60 years old. More than half of the sampled patients (2.9%) were male, 53.8% had graduated from junior middle school or primary school, and 62.2% had an annual income < ¥50,000. Most (91.7%) were married, 64.1% were employed, and 50.2% were living in rural areas. One-third (31.2%) of the participants had been diagnosed with hypertension for at least 10 years, 57.4% had a family history of hypertension, 30.2% had developed complications of hypertension, 64.3% were taking only one antihypertensive drug, and only 8.6% lived alone or with one person.

**Table 1:** Patient characteristics (n = 420).

Items	Group	N	%
Age (years)*	22–44	39	9.3%
	45–59	131	31.2%
	60–88	250	59.5%
Gender	Male	222	52.9%
	Female	198	47.1%
Education level	Primary and below	121	28.8%
	Junior middle school	105	25.0%
	High school	100	23.8%
	Junior College	60	14.3%
	College degree and above	34	8.1%
Annual household income Chinese RMB (¥)	<10,000/year	57	13.6%
	10,000–29,999/year	83	19.8%
	30,000–49,999/year	121	28.8%
	50,000–99,999/year	75	17.9%
	≥100,000/year	84	20.0%
Marital status	Married	385	91.7%
	Unmarried	8	1.9%
	Divorced or widowed	27	6.4%
Occupational status	Employed	269	64.1%
	Retired	110	26.2%
	Unemployed	41	9.8%
Registered residence	Urban	209	49.8%
	Countryside	211	50.2%
Duration of hypertension	<3 years	94	22.4%
	3–4.9 years	77	18.3%
	5–9.9 years	118	28.1%
	≥10 years	131	31.2%
Family history of hypertension	Yes	241	57.4%
	No	179	42.6%
Hypertension complication	Yes	127	30.2%
	No	293	69.8%
Number of prescribed antihypertensive drugs	One	270	64.3%
	2–3	132	31.4%
	≥4	18	4.3%
Number of co-lived person	1 or 0	36	8.6%
	2–4	250	59.5%
	5–7	105	25.0%
	≥8	29	6.9%

*The mean for age was 60.6 years with a standard deviation of 12.4.

### Responses to the Chinese Medication Literacy Scale for Hypertensive Patients

Medication literacy scores ranged from 0 to 37 on the C-MLSHP, and the mean score for the medication literacy scale was 24.03 (SD = 5.13) (**Table 2**). Compared with the full score of 37, the medication literacy level was suboptimal. Additionally, the mean scores for each dimension for medication literacy were 0.69 (SD = 0.25) for the knowledge dimension, 0.63 (SD = 0.14) for the attitude dimension, 0.64 (SD = 0.32) for the skill dimension, and 0.64 (SD = 0.15) for the behavior dimension. Medication literacy in the knowledge dimension for hypertensive patients was higher than that in the other dimensions.

**Table 2:** Scores for the overall level of medication literacy and for each dimension (n = 420).

Dimensions	Total score	Lowest score	Highest score	Mean ± SD of each dimension	Mean ± SD of each item
Knowledge literacy	9	1	9	6.22 ± 2.22	0.69 ± 0.25
Attitude literacy	8	2.5	7.75	5.04 ± 1.16	0.63 ± 0.14
Skill literacy	7	0	7	4.50 ± 2.21	0.64 ± 0.32
Behavior literacy	13	3	12.75	8.27 ± 1.90	0.64 ± 0.15
Total score	37	11.5	34	24.03 ± 5.13	0.65 ± 0.14

### Responses to the C-MMAS-8

Adherence scores ranged from 0 to 8 on the C-MMAS. The mean score for the medication adherence scale was 4.82 (SD = 2.11). Based on the MMAS-8 score, patients were categorized into three groups as described in the Materials and Methods section: low adherence (MMAS-8 score < 6), moderate adherence (MMAS-8 score ≥ 6 to < 8), or high adherence (MMAS-8 score 8). Therefore, the results indicated that more than half of the respondents (63.6%) had low adherence, 121 (29.5%) had moderate adherence, and only 32 (7.6%) had high adherence. Using the cut-off point of 6 on the MMAS-8 and for the purpose of this analysis, the study population comprised 63.6% poor adherers and 37.1% good adherers. Responses for each of the MMAS-8 are summarized in **Table 3**.

**Table 3:** Responses for each question in the (MMAS-8) scale. (Total study population = 420).

Items	Answered “yes”	%
1. Do you sometimes forget to take your pills?	212	50.5%
2. People sometimes miss taking their medications for reasons other than forgetting. Thinking over the past 2 weeks, were there any days when you did not take your medicine?	141	33.6%
3. Have you ever cut back or stopped taking your medication without telling your doctor, because you felt worse when you took it?	140	33.3%
4. When you travel or leave home, do you sometimes forget to bring along your medication?	205	48.8%
5. Did you take your medicine yesterday?	84	20.0%
6. When you feel like your illness is under control, do you sometimes stop taking your medicine?	336	80.0%
7. Taking medication every day is a real inconvenience for some people. Do you ever feel hassled about sticking to your antihypertensive treatment plan?	188	44.8%
8. How often do you have difficulty remembering to take all your medications?		
All the time	239	56.9%
Usually	15	3.6%
Sometimes	27	6.4%
Once in a while	110	26.2%
Never/rarely	141	33.6%
Overall score	127	30.2%

### Correlation Between Medication Literacy and Medication Adherence

**Figure 1:**
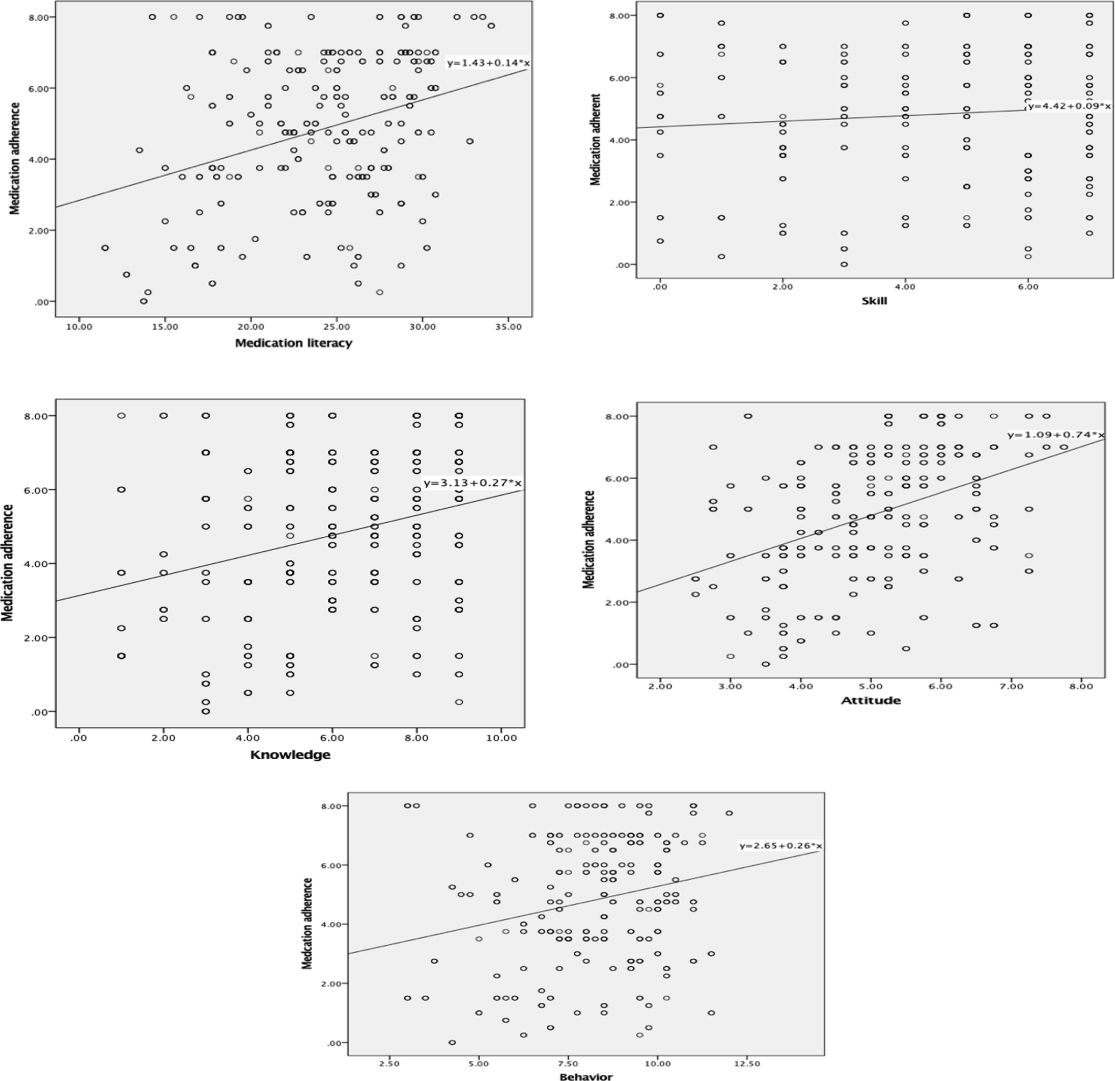
Graph 1–5 correlation between medication literacy and its domains and medication adherence.

**Table 4** and **Figure 1** show the results of the correlation between medication literacy and medication adherence. Medication literacy was positively correlated with medication adherence (r = 0.342, P < 0.01). For each dimension of medication literacy, three dimensions of medication literacy—namely, knowledge (r = 0.284, P < 0.01), attitude (r = 0.405, P < 0.01), and behavior (r = 0.237, P < 0.01)—were positively associated with adherence, though the association with knowledge may be weak. In addition, the dimension of skill literacy was not found to be associated with adherence at a significant level.

**Table 4:** Correlation between hypertensive patients’ medication literacy and medication adherence (n = 420).

	Knowledge literacy	Attitude literacy	Skills literacy	Behavior literacy	Total score of medication literacy
Medication adherence	0.284^**^	0.405**	0.093	0.237^**^	0.342^**^

**P values < 0.01 were considered statistically significant (two-tailed).

### Factors Predicting Medication Adherence

Variables of patient characteristics and four dimensions of medication literacy—namely, knowledge, attitudes, skills, and behavior—were included in the binary logistic regression analysis as independent variables. For the purpose of analysis, patients were classified into two (poor adherence and good adherence) rather than three categories (low, moderate, high) based on MMAS-8 scores: poor adherence (MMAS-8 score < 6) and good adherence (MMAS-8 score ≥ 6). **Table 5** shows the results of the binary logistic regression analysis identifying significant factors that predict medication adherence. The model can explain 15.8% (21.6%) of the change in the adherence level. Significant factors that were independently associated with adherence to hypertensive medication were as follows: attitude literacy [OR 2.174 (95% CI: 1.748–2.706); P = 0.000], behavior literacy [OR 1.139 (95% CI: 1.002–1.294); P = 0.046], and annual income [OR 1.199 (95% CI: 1.011–1.421); P = 0.037]. Individuals with better attitude and behavior literacy as well as higher annual income were more likely to be adherent to antihypertensive drugs. **Table 6** shows the independent variables assignment of binary logistic regression analysis of hypertensive patients’ medication adherence.

**Table 5:** Binary logistic regression analysis for factors predicting medication adherence.

Variables	Adherent/nonadherent				
	β	SE	Odds ratio	95% CI	P-value
Attitude	0.130	0.065	2.174	1.748, 2.706	0.000
Behavior	0.777	0.111	1.139	1.002, 1.294	0.046
Annual income	0.181	0.087	1.199	1.011, 1.421	0.037

**Table 6:** Independent variables assignment of binary logistic regression analysis of hypertensive patients’ medication adherence.

Independent variables	Assignment
Gender	Male = 1; female = 2
Age group	18–45 = 1; 45–60 = 2; ≥60 = 3
Marital status	Take ‘unmarried’ as reference of dummy variable: M1 = married (摬1摬 摬0摬), M2 = divorced or widowed (摬0摬 摬1摬)
Level of education	Primary or below = 1; Junior middle school = 2; senior high school or secondary specialized school = 3; junior college = 4; bachelor degree or above = 5
Occupational status	Take ‘retired’ as reference of dummy variable; P1 = employed (摬1摬 摬0摬), P2 = unemployed (摬0摬 摬1摬)
Annual household income Chinese RMB (¥)	<10,000/year = 1; 10,000–29,999/year = 2; 30,000–49,999/year = 3; 50,000–99,999/year = 4; ≥100,000/year = 5
Registered residence	City = 1; rural = 2
The number of co-lived person	1 or 0 = 1; 2–4 = 2; 5–7 = 3; 8 or more = 4
Duration of hypertension	<3 years=1; 3–4.9 years = 2; 5–9.9 years = 3; ≥10 years = 4
family history of hypertension	Yes = 1; none = 2
Hypertension complications	Yes = 1; none = 2
Number of prescribed antihypertensive drugs	one = 1; 2–3 kinds = 2; 4 or more = 3
Knowledge literacy	Continuous value
Attitude literacy	Continuous value
Skill literacy	Continuous value
Behavior literacy	Continuous value
Medication literacy	Continuous value

## Discussion

The aim of the current study was to determine the association between medication literacy and medication adherence as well as the extent of medication adherence among adult hypertensive patients in several hospitals and healthcare services in China. Medication literacy, as the crucial indicator of safe medication use (Pouliot et al., 2018), should be assessed among hypertensive patients. Hypertensive patients usually have uncontrolled blood pressure due to poor adherence to antihypertensive drugs (Morisky et al., 2008; Abegaz et al., 2017; Adisa et al., 2018; Beune et al., 2019). Identifying patient groups that are more likely to be adherent and determining whether medication literacy is the predicting factor of medication adherence are pivotal to designing targeted management strategies. The present study was designed to build on prior studies that have documented poor adherence and blood pressure control among patients in China and other countries by identifying factors affecting medication adherence (Yan et al., 2014; Adisa et al., 2018; ; Rampamba et al., 2018; Beune et al., 2019; Fatani et al., 2019).

In the current study, the mean score for the medication adherence scale was 4.82 (SD = 2.11), which was not good compared with the cutoff point of 6 for differentiating poor and good adherence and was lower than the mean adherence score of 5.33 (SD = 2.49) reported by Pakistan (Bashir et al., 2018). It was found that more than half of the sampled hypertensive patients (63.6%) had low levels of medication adherence, which was higher than an international pooled percentage of 31.1% with the MMAS-8 (Uchmanowicz et al., 2019). Poor adherence to antihypertensive medication not only is associated with poor blood pressure control but also accelerates the development of hypertension-related complications and increases the rate of hospital admissions (Sabaté, 2003; Abegaz et al., 2017; Jafar et al., 2018; Beune et al., 2019; Xue et al., 2019). In accordance with the findings of other studies in the mainland of China, the rate of poor medication adherence across China was 64.8%, with values as high as 78.7% (Kang et al., 2015; Wong et al., 2015; Hou et al., 2016; Ma, 2016; Pan et al., 2017), indicating that adherence to drug therapy for Chinese hypertensive patients is a great concern and needs greater attention. The rate of poor adherence of hypertensive patients in Hong Kong was 44.9% (Rampamba et al., 2018), which was slightly lower than that in the mainland of China. This might be due to the better healthcare system and more developed economy in Hong Kong. Similarly, in other countries, the majority of hypertensive patients had poor or non-adherence, with levels ranging from 54% to 55.9% (Lo et al., 2016; Khayyat et al., 2017). Furthermore, only 6.2% and 8.9% of patients in Saudi Arabia and Nigeria exhibited high adherence to medication treatment (Adisa et al., 2018; Fatani et al., 2019). The difference in the adherence rates between the literature and the current study may be related to the differences in the study population, patients’ knowledge, health literacy, and the complexity of patients’ regimens and health conditions. Distinctively, 81.7% of hypertensive patients had good adherence in a Korean study (Choi et al., 2018), which might be attributed to the different populations and ways of measuring adherence. In this Korean study, the pill count method was used to measure adherence, while the majority of other studies applied self-report scales. Disparities among study results caused by varying adherence measures were also confirmed in a meta-analysis of medication adherence (Nielsen et al., 2017).

Medication literacy was measured by a self-developed C-MLSHP, which is a self-report scale. The mean score for the medication literacy scale was 24.03 (SD = 5.13). Compared to a full score of 37, the medication literacy level for hypertensive patients was sub-optimal and still needs to be improved. Considering the varying score distribution for the four dimensions of this medication literacy scale, it is not applicable for this scale to have a specific cutoff point for distinguishing good from poor medication literacy or even high, medium, and low levels. Since a national norm for standardization for medication literacy level nationwide in China has not been established due to the limited sample size in the present study, we had to explain the medication literacy score by comparing it with the full score of 37. Higher scores closer to 37 indicate a higher level of medication literacy. However, it is a relative comparison such that patients might have unbalanced scores among the dimensions of medication literacy; therefore, we cannot identify a patient as having a sufficient medication literacy level even when the patient had full scores for attitude and behavior literacy but low scores for knowledge and skill literacy. Considering the specific scores for medication literacy and its four domains, it is suggested that the levels of medication literacy and its four domains still need to improve (Zhong et al., 2016; Pouliot et al., 2018).

In consistent with the present study, an insufficient medication literacy level has been found in several studies of Chinese populations (Zhong et al., 2016; Zheng et al., 2017), though different populations were studied and varying scales were applied for the medication literacy measure. There is a lack of hypertension-specific medication literacy assessment studies worldwide, but health literacy has been widely studied, and direct and mediating associations with adherence and blood pressure control in hypertensive patients have been determined (Nafradi et al., 2016; Wannasirikul et al., 2016). Medication literacy is the manifestation of health literacy in the context of medication use, and inappropriate medication use was identified to be significantly associated with low medication literacy levels (Lee et al., 2017). In the current study, medication literacy was found to be positively correlated with adherence for hypertensive patients (r = 0.342, P < 0.01). Therefore, patients’ ability to use appropriate and safe medications should be improved by targeting medication literacy to some extent.

Specifically, it was found that three dimensions of medication literacy—namely, knowledge, attitudes, and behavior literacy—had a significantly positive correlation with medication adherence, though the correlations were weak. This might be due to the limited sample size in the present study, although it was based on a correct sample size calculation. Knowledge refers to whether patients know about hypertension, antihypertensive therapy, adverse effects of antihypertensives, etc. However, it was notable that the dimension of knowledge only showed a relatively weak correlation with medication adherence (r = 0.284, P < 0.01). In addition, it was found that patients’ education level and the knowledge dimension of medication literacy were not independent factors predicting adherence in hypertensive patients. These results contrasted with those of other studies worldwide (Boima et al., 2015; Pan et al., 2017; Rampamba et al., 2017), in which knowledge of hypertension and medication therapy was positively associated with medication adherence. One explanation might be the different methods of knowledge measurement. Another possible reason may relate to the different features of the older Chinese population compared with that in other countries; the majority of individuals in this population are limited in their educational background and knowledge level, but they are prone to trusting physicians about their treatment. It was also found in the present study that the attitude literacy level was relatively high in patients over 60 years old, although they had a low education level and limited knowledge. This result might also be explained by self-perceptions of ageing (Hou et al., 2016). Considering this contradiction, further studies might still be needed to elucidate the interaction between knowledge and adherence. Interestingly, a prior study indicated that the least knowledgeable patients showed good adherence and, that as knowledge increased, adherence somehow decreased to moderate or poor (Nurhussein et al., 2018). Likewise, individuals who had not received any form of formal education (Boima et al., 2015) or those who were illiterate (Nurhussein et al., 2018) were more likely to show adherence. The reason for these phenomena might be that educated participants were more skeptical of the use of antihypertensives (Boima et al., 2015).

In our study, the dimensions of attitude and behavior in medication literacy as well as annual incomes for hypertensive patients were found to be independent predictors associated with medication adherence. A range of studies have examined patients’ attitudes toward hypertension and their medication treatment or health beliefs about hypertension in relation to adherence, and the results were consistent with our study (Boima et al., 2015; Kang et al., 2015; Al-Noumani et al., 2018). Fewer concerns about medication (Boima et al., 2015), stronger and positive beliefs about hypertension, self-perceived disease severity, and medication necessity (Lo et al., 2016; Jande et al., 2017; Al-Noumani et al., 2018), as well as good self-perceived health status (Kang et al., 2015) were found to be associated with higher adherence. Adequate knowledge combined with correct and positive attitudes toward hypertension is the most fundamental premise for patients to adhere to medication therapy. Therefore, targeted strategies implemented to improve medication adherence should incorporate knowledge and beliefs as key components. Patient education and counseling regarding knowledge about hypertension as well as the necessity and side effects of medications are important to maximize patients’ knowledge and positive beliefs about hypertension so as to improve their medication adherence (Al-Noumani et al., 2018).

Skills literacy refers to patients’ abilities to read, comprehend, and calculate drug dosage, frequency, or physician consultation periods, appropriate medication storage, and correct ways to cope with adverse drug effects and suboptimal medication effectiveness. In our study, skills literacy was neither correlated with adherence nor found to be the significant factor predicting adherence. In contrast to the results of the present study, the inability to take medication correctly might be a particularly important barrier to medication adherence (Foster et al., 2018). Individuals with lower reading comprehension ability were more likely to have uncontrolled blood pressure (Shibuya et al., 2011). The discrepancy between two different results might be due to variance in skill measurement; further research has to be conducted to test the current results. Prior studies have found that the effect of health literacy involving skill measurement items on medication adherence was mediated by adherence self-efficacy (Nafradi et al., 2016; Wannasirikul et al., 2016). However, there have not been any studies specifying the association between the skills domain of medication literacy and medication adherence. Thus, further studies should be conducted to specify and determine the interactions between the skills domain of medication literacy and adherence.

Behavior literacy refers to the ability to seek information about hypertension, observe adverse reactions and feedback, monitor blood pressure, and make clinic visits to physicians.

Higher behavior literacy was significantly associated with good adherence to hypertensive agents, with similar results reported by several studies (De Albuquerque et al., 2018); people who attended between four and six nursing consultations had better adherence. Studies found that forgetfulness was the most common reason for nonadherence (Iloh et al., 2013; Adisa et al., 2018), which was consistent with our study results. In addition, reminder systems (Gwadry-Sridhar et al., 2013) and FDCs (Du et al., 2018) have proved to be conducive to good adherence by helping patients stick to persistent medication-taking behavior. However, few studies have focused on the association between the behavior domain of medication literacy and medication adherence. Future studies on this topic must be carried out. This study found that the total score for medication literacy was positively but weakly correlated with adherence, but in the binary logistic regression analysis, the variable of medication literacy did not enter the final model, which means that the total score for medication literacy was not associated with adherence. However, according to the specific data, one possible explanation we suspect was that there must be a mediator between medication literacy and adherence. Further studies have to be carried out to identify and clarify the mechanism of this interaction. Therefore, we still hold that medication literacy was associated with adherence.

Another predictor of adherence found in the present study was patients’ annual income, as patients with higher annual income were more likely to be adherent to antihypertensive drugs. Several studies have also come to similar results (Lemstra and Alsabbagh, 2014; Ma, 2016; Williams et al., 2018), and patients with lower income tend to have reduced antihypertensive drug use due to costs.

Age, gender, marital status, occupational status, education level, number of prescribed antihypertensive drugs, family history of hypertension, hypertension duration, and comorbidities have been proved to be associated with adherence to hypertensive agents, in contrast to the current study. Registered residence in urban and rural areas and the number of persons in the household were not associated with adherence in the present study or in other studies. These differences might be caused by variations in the study populations.

## Strengths and Limitations

Strengths: first, validated questionnaires of the C-MMAS and medication literacy scale with good reliability and validity were used to ensure that the data collected in this study were valid. Second, the stratified cluster sampling method was used to make the sample population relatively balanced in demographic characteristics. This method helps to neutralize the influence of hospital rankings on patient characteristics. Third, this research was the first time that the association between medication literacy and medication adherence was studied and identified, which offers insight into improving hypertensive patients’ adherence to treatment.

Limitations: first, only one self-report adherence measurement tool was used; therefore, the results for patients’ medication adherence were subjective and might not be convincing. Future studies might incorporate some objective measures of adherence, such as pill count methods and biochemistry indicators. Second, this study was conducted in one city of China; therefore, our results may not be representative. Further studies including a larger sample and more areas should be carried out. Most of our participants were older persons (≥45 years); thus, we obtained limited information about medication literacy and adherence for younger patients under 45 years old, to whom the conclusions of our study might not be generalized. Future studies should be conducted with a more age-balanced population.

## Conclusion and Implications

In summary, hypertensive patients’ medication literacy and medication adherence still need to be improved. Medication literacy had a positive relationship with medication adherence. Two dimensions of attitude and behavior in medication literacy as well as annual income were significant predictors associated with adherence to hypertensives. Promotion strategies targeting the attitude and behavior dimensions of medication literacy should be incorporated into education and counseling programs with healthcare professionals. Based on our study, future studies should be carried out to further test the effects of the knowledge and skill dimensions of medication literacy on adherence.

## Ethics Statement

This study was carried out in accordance with the recommendations of “Ethics Committee of the Third Xiangya Hospital” with written informed consent forms from all subjects. All subjects gave written informed consent forms in accordance with the Declaration of Helsinki. The protocol was approved by the “Ethics Committee of the Third Xiangya Hospital.”

## Author Contributions

ZZ instructed the whole study design and is responsible for the whole project. SS and ZS were in charge of the paper writing and data collection. YD and SD made contributions to the statistical analysis and data collection.

## Funding

The authors would like to thank the National Natural Science Foundation of China (Project number: 71603290) for granting and supporting this work.

## Conflict of Interest Statement

The authors state that there are no other contributors to this study. Additionally, the authors declare no potential conflicts of interest with respect to the authorship and publication of this article.
